# Acquired Hemophilia A: A Case Report on a Rare Disease Manifesting as Persistent Hematuria

**DOI:** 10.7759/cureus.35763

**Published:** 2023-03-04

**Authors:** Amber Kuta, Karina S Shendrik, Linda Youn, Alena Yarema

**Affiliations:** 1 Department of Internal Medicine, St. Bernards Medical Center, Jonesboro, USA

**Keywords:** hemophilia a, prolonged activated partial thromboplastin time, acquired coagulation disorders, factor viii and factor viii inhibitors, gross hematuria

## Abstract

Hemophilia A is most commonly a genetic clotting disorder that is caused by a decrease or lack of activity of clotting factor VIII. Acquired hemophilia, a rarer subset can occur later on in life. The incidence rate of the latter subtype is estimated to affect one per million cases a year. Given the rarity of the disease, the associated hemoglobin and hematocrit reduction seen from this can easily be missed and attributed to concomitant disorders such as hematuria. Our patient initially presented with persistent hematuria, was treated with multiple rounds of antibiotics, and underwent a plethora of urological studies with no resolution or explanation of her symptoms. It wasn't until her ICU admission that coagulation studies were reviewed in depth and workup for hemophilia A began. The American Urologic Association's hematuria workup is currently limited to the genitourinary system with no consideration for hematological disorders. We believe that the medical community may benefit from further research in this area in order to avoid mismanaging patients medically and thus minimize morbidity and mortality.

## Introduction

Acquired factor VIII inhibitor (FVIII) also known as acquired hemophilia A (AHA) is a bleeding disorder caused by autoantibodies against coagulation factor VIII, inhibiting its activity in the coagulation cascade. FVIII is a cofactor for factor IXa that leads to the activation of factor X (FX) in the intrinsic pathway; without FX activation, thrombin generation is also inhibited. This inhibition leads to patients that present with new-onset bleeding, most commonly mucocutaneous, who lack a personal or family history of bleeding conditions [1]. Mucosal bleeding occurring within the genitourinary system presents as hematuria. Hematuria as a symptom manifests among many varied pathologies. This makes the differentials broad and certain diagnoses overlooked, often causing substantial diagnostic challenges for physicians. Our case represents an unequivocal delay in diagnosis and treatment due to the complexity of unveiling a rare disease presenting as a common symptom.

## Case presentation

Our patient was a 71-year-old nursing home resident with a significant past medical history of advanced dementia who first presented to our hospital after being transferred from an outside facility for urological evaluation after being found to have gross hematuria. Of note, history was obtained from nursing home staff and the patient's daughter, as the patient was a poor historian due to advanced dementia. At the outside hospital, labs were notable for hemoglobin of 8 g/dL, hematocrit of 24.3%, prothrombin time (PT) of 10.0 sec, international normalized ratio (INR) of 0.95, and activated partial thromboplastin time (APTT) of 63 sec. Prolonged APTT was not addressed at that time. A urinalysis (UA) was done showing a large amount of blood, 21-50 red blood cells (RBC)/high power field (HPF), white blood cells (WBCs), >20/HPF red blood cells (RBCs), moderate leukocyte esterase, positive nitrates with 2+ bacteria. The patient completed a two-week course of levofloxacin 15 days prior to this admission secondary to a presumed urinary tract infection (UTI). The patient was then started on ceftriaxone for the suspected infection seen on repeated urinalysis. Urology was consulted for gross hematuria and they consequently performed a cystoscopy, which showed no pathology in the bladder. A computed tomography (CT) scan revealed no kidney or ureter pathology. It was concluded that hematuria was secondary to infection and the patient was discharged.

Approximately one month later, the patient returned and was admitted to the intensive care unit on account of hypotension, profound anemia, continued hematuria, and increased ecchymosis. Initial hemoglobin was 3.5 g/dL, hematocrit was 12.8%, and platelet count was 306^3/uL. Coagulation studies were obtained and initially showed a PT of 12.6 sec, INR of 1.1, and APTT of 145.6 sec. After a review of the patient’s home medications, 81 mg aspirin was discontinued due to prior recurrent episodes of hematuria. The patient was never on any other antiplatelets or anticoagulants. There were no signs of gastrointestinal (GI) bleeding and the CT scan did not show any source of internal blood loss. The patient’s hemoglobin and hematocrit levels responded appropriately to the initial two units of packed red blood cells (PRBC). The Hematology/Oncology service was consulted and initiated a coagulation workup. A mixing study, lupus anticoagulant, as well as factor VIII, IX, and XI inhibitor levels, were ordered. Lupus anticoagulant testing was negative. Iron levels were also noted to be low and the patient was started on intravenous (IV) iron. On hospital day seven, the patient continued to have hematuria with normal PT/INR and persistently prolonged PTT at 130 sec. Mixing studies results showed uncorrected clotting time, suggesting the presence of an inhibitor. Heparin-induced thrombocytopenia (HIT) antibody testing was negative. Our factor VIII inhibitor testing showed a factor VIII inhibitor level (Bethesda) of 846 and factor VIII activity was measured at less than one.

The patient continued to have hematuria requiring a total of 12 units of leukocyte-reduced RBC along with two units of fresh frozen plasma during that hospital stay. Given that factor VIII inhibitor bypass activity (FEIBA) is unavailable at our facility, we made a request for a transfer to a tertiary facility. Due to the patient's ongoing blood loss and declining hemoglobin, recombinant factor VIIa was administered, after which the patient's PTT decreased to 112 seconds; however, hematuria did not resolve. The patient was then successfully transferred to a tertiary facility in anticipation of further management with FEIBA.

## Discussion

Hematuria, defined as red blood cells in the urine, can be divided into gross hematuria and microscopic hematuria. Gross hematuria is the presence of blood in urine that is visible to the naked eye. Microscopic hematuria is blood in the urine detected by testing such as urinalysis or urine microscopy. It can be further divided into asymptomatic or symptomatic. A detailed history should be obtained that includes but is not limited to the characterization of current hematuria and associated symptoms, previous episodes of hematuria, a family history of hematuria, procedural history, smoking history, current medications, and recreational drug use. A physical exam should be performed to assist with forming a comprehensive list of differential diagnoses. It is helpful to divide potential causes into glomerular vs non-glomerular pathology. Evaluation should start with a urinalysis (UA) over urine dipstick due to the increased sensitivity and specificity of the test resulting in fewer false-positive or false-negative findings. If urinalysis shows the presence of three or more RBCs per HPF, that is considered microscopic hematuria. A UA that shows significant WBCs is positive for leukocyte esterase, and nitrites are suggestive of a urinary tract infection. Glomerulonephropathy should be considered if excessive proteins are present in the urine. Urine microscopy can be used to differentiate glomerular and non-glomerular bleeding by looking for RBC morphology and RBC casts. If dysmorphic RBCs and RBC casts are present then a kidney biopsy should be pursued. Additional labs to rule out acute kidney injury should be performed. Imaging can be helpful in diagnosis and usually begins with an ultrasound of the kidneys, ureters, and bladder. A CT scan of the abdominopelvic region with or without contrast or even magnetic resonance imaging (MRI) of the region can be useful if the CT scan was not possible or did not provide the necessary information. If the workup thus far has been negative then urine cytology and cystoscopy should be done to further evaluate hematuria [2].

With over two million patient referrals to urology each year in America, hematuria is considered relatively common. The American Urology Association (AUA) updated their guidelines in 2020 for the evaluation of hematuria. The current algorithm for microhematuria starts with a UA with microscopy that shows greater than or equal to three RBCs/HPF followed by history and physical exam. If the history and physical exam leads the investigator to believe that there is a non-malignant or gynecologic source of the hematuria then those should be identified or ruled out before proceeding with further evaluation [3].

Once treatment of the non-malignant or gynecologic sources has been completed then a urinalysis should be repeated. Further evaluation by risk stratification is required if repeat studies are positive or non-malignant, gynecologic sources are ruled out, or thorough history and physical exam do not lead one to believe that there is a non-malignant or gynecologic source behind the hematuria. Risk stratification can be divided into low risk, intermediate risk, or high risk. Risk stratification is based on the patient's sex, age, smoking history, amount of RBCs/HPF on one UA, prior episodes of gross hematuria, and additional risk factors for urothelial cancer. Based on the level of risk, further workup with either repeat urinalysis within six months, renal ultrasound, cystoscopy, CT urogram, or a combination of these is recommended. If it is decided to repeat UA within six months or the above-mentioned modalities do not reveal a pathology then repeat UA within 12 months can be considered. If pathology is found then it should be treated as indicated followed by a repeat UA. If the UA within 12 months or the repeat UA after treatment is still positive for hematuria then shared decision-making regarding repeat evaluation vs. observation should be considered. Re-evaluation should be done if a patient develops gross hematuria, has an increase in microhematuria, or if there are new urologic symptoms [3]. Although hematuria is a common symptom, coagulopathies should still be considered a potential etiology.

Hemophilia A is typically a deficiency of factor VIII that is genetic; however, patients may develop inhibitors to factor VIII later in life, which is considered acquired hemophilia A. These individuals commonly have immunoglobulin G (IgG) autoantibodies that bind FVIII and exhibit second-order kinetics leading to incomplete factor VIII inactivity. The amount of remaining measurable activity gives minimal, if any, protection against hemorrhage. The incident rate of acquired inhibitors/autoantibodies in non-hemophiliacs is about one case per million per year. As stated previously, patients typically present with new-onset bleeding without a personal or familial history of bleeding, disorders with mucocutaneous bleeding being the most common subtype [1].

Acquired hemophilia A coagulation studies reveal a normal PT and thrombin time (TT) with a prolonged APTT. The differential diagnosis for a prolonged APTT should include a deficiency of coagulation factor(s) (e.g. factor VIII, factor IX, factor XII, prekallikrein, or high-molecular-weight kininogen), heparin contamination, factor inhibitor (e.g. FVIII), and presence of a non-specific inhibitor (e.g. lupus anticoagulant). Prolongation of APTT beyond 100 seconds is atypical for FVIII inhibitors. Alternative or co-existing causes, such as the presence of heparin use, lupus anticoagulant (LA), or deficiency of a coagulation contact factor, should be considered. The typical initial study for prolonged APTT is a mixing study in which an equal volume of normal plasma is mixed with the patient’s plasma to determine if the APTT is corrected or not. If not corrected then additional testing should be performed. It is important to rule out nonspecific inhibitors, such as heparin contamination and lupus anticoagulant, as these are the two most common causes of a prolonged aPTT [1,4].

## Conclusions

Hematuria is a common presenting symptom seen in multiple care settings. Initial workup typically includes an investigation of infectious etiologies, imaging, and a urology consultation to rule out urological conditions; however, less common diseases should also be considered. In our patient, her initial presentation showed only hematuria and she underwent two courses of antibiotics and urologic evaluation while her elevated APTT was overlooked and not addressed. It was not until the patient came in with profound anemia, multiple ecchymoses, and continued hematuria, requiring intensive care, that her coagulation abnormality was considered. The American Urological Association does not include coagulation studies as part of the current initial evaluation of hematuria. The benefit of early coagulation studies in evaluating patients with hematuria should be further explored. As this is a single case report, further studies are needed to determine a broader clinical significance. We urge physicians to broaden their differential diagnoses and think unconventionally to prevent delayed diagnoses and improve future outcomes.
